# Complete mitochondrial genomes reveal robust phylogenetic signals and evidence of positive selection in horseshoe bats

**DOI:** 10.1186/s12862-021-01926-2

**Published:** 2021-11-03

**Authors:** Lin Zhang, Keping Sun, Gábor Csorba, Alice Catherine Hughes, Longru Jin, Yanhong Xiao, Jiang Feng

**Affiliations:** 1 Jilin Provincial Key Laboratory of Animal Resource Conservation and Utilization, Northeast Normal University, Changchun, 130117 China; 2 Key Laboratory of Vegetation Ecology, Ministry of Education, Changchun, China; 3 Department of Zoology, Hungarian Natural History Museum, Budapest, Hungary; 4 Centre for Integrative Conservation, Xishuangbanna Tropical Botanical Garden, Chinese Academy of Sciences, Menglun, Mengla County, 666303 Yunnan China; 5 College of Life Science, Jilin Agricultural University, Changchun, 130118 China

**Keywords:** Mitogenome, *Rhinolophus*, Comparative analysis, Positive selection

## Abstract

**Background:**

In genus *Rhinolophus*, species in the *Rhinolophus philippinensis* and *R. macrotis* groups are unique because the horseshoe bats in these group have relatively low echolocation frequencies and flight speeds compared with other horseshoe bats with similar body size. The different characteristics among bat species suggest particular evolutionary processes may have occurred in this genus. To study the adaptive evidence in the mitochondrial genomes (mitogenomes) of rhinolophids, especially the mitogenomes of the species with low echolocation frequencies, we sequenced eight mitogenomes and used them for comparative studies of molecular phylogeny and adaptive evolution.

**Results:**

Phylogenetic analysis using whole mitogenome sequences produced robust results and provided phylogenetic signals that were better than those obtained using single genes. The results supported the recent establishment of the separate *macrotis* group. The signals of adaptive evolution discovered in the *Rhinolophus* species were tested for some of the codons in two genes (*ND2* and *ND6*) that encode NADH dehydrogenases in oxidative phosphorylation system complex I. These genes have a background of widespread purifying selection. Signals of relaxed purifying selection and positive selection were found in *ND2* and *ND6*, respectively, based on codon models and physicochemical profiles of amino acid replacements. However, no pronounced overlap was found for non-synonymous sites in the mitogenomes of all the species with low echolocation frequencies. A signal of positive selection for *ND5* was found in the branch-site model when *R. philippinensis* was set as the foreground branch.

**Conclusions:**

The mitogenomes provided robust phylogenetic signals that were much more informative than the signals obtained using single mitochondrial genes. Two mitochondrial genes that encoding proteins in the oxidative phosphorylation system showed some evidence of adaptive evolution in genus *Rhinolophus* and the positive selection signals were tested for *ND5* in *R. philippinensis*. These results indicate that mitochondrial protein-coding genes were targets of adaptive evolution during the evolution of *Rhinolophus* species, which might have contributed to a diverse range of acoustic adaptations in this genus.

**Supplementary Information:**

The online version contains supplementary material available at 10.1186/s12862-021-01926-2.

## Background

Mitochondria are the energy metabolism centers that play critical roles in adenosine triphosphate (ATP) synthesis and heat generation [1]. The oxidative phosphorylation system (OXPHOS), which is the main pathway that produces cellular energy via ATP, provides approximately 95% of the energy required by an organism for the basic activities of life [2–4]. There are five complexes in OXPHOS; four of these complexes (I, III, IV, and V) are co-encoded by nuclear and mitochondrial genes, whereas complex II is encoded exclusively by nuclear genes [2, 5, 6]. Vertebrate mitochondrial genomes (mitogenomes) comprise a large non-coding region (control region) and 37 genes; 13 encode OXPHOS proteins, two encode ribosomal RNAs (rRNAs), 22 encode transfer RNAs (tRNAs) [7, 8].

Mitogenomes are characterized by their small size, lack of recombination, maternal inheritance, and high substitution rates. All these properties have made mitochondrial genes ideal for used in phylogeography, species identification, and molecular phylogenetics [1, 9–12]. Mitochondrial genes are linked within the same DNA molecule, so it is expected that different mitochondrial genes share the same genealogy [9]. However, incongruent phylogenetic results have frequently been found among different mitochondrial genes, indicating these genes contain different phylogenetic signals [13–15]. Although single or a few concatenated genes have often been used to represent an entire mitogenome, the best genes or regions for a particular study are highly taxa-dependent [9, 16]. Analyses of mitogenomes have proven useful for inferring recent evolutionary relationships.

Mitogenome have historically been assumed to evolve neutrally. However, the 13 mitochondrial protein-coding genes (PCGs) are expected to have experienced non-neutral changes because of their functional importance in OXPHOS [17, 18]. Mitochondria function to supply cellular energy and produce heat and are therefore extremely sensitive to heat and energy-related selective pressure [1, 19–22]. The role of selection in the evolution of mitochondrial DNA (mtDNA) attracted little attention until recently [3, 9, 23, 24]. Mitochondrial OXPHOS genes have been shown to display evidence of adaptive evolution in, for example, dogs, bats, birds and fishes [21, 22, 25, 26], and numerous studies have detected signals of positive selection in mitochondrial genes [21–23, 27], suggesting positive selection plays a strong potential role in adaptation [28, 29].

Bats (order Chiroptera) are unique mammal that have developed flight as their primary locomotion mode [21, 30]. Bats have undergone unique adaptive evolution for their survival and the evolution of flight is an energetically expensive adaptation. It is therefore likely that genes involved in energy metabolism would have undergone much evolutionary change. Shen et al. [21] resequenced 77 OXPHOS genes, including mitochondrial- and nuclear-encoded OXPHOS genes from four species of bats. Their results showed adaptive evolution of genes involved in energy metabolism specific on the common ancestral bat lineage [21]. Mitochondrial PCGs in particular have vital roles in energy production by directly mediating proton-pumping across the membrane [10]. Analyses of these PCGs may greatly facilitate the understanding of chiropteran evolution [30]. Complete mitogenome data are available for only 99 bat species, which is a small proportion of the ~ approximately 1300 bat species [31]. Therefore, the generation and analysis of additional complete mitogenome sequences will help to further understand bat evolution.

**Fig. 1:**
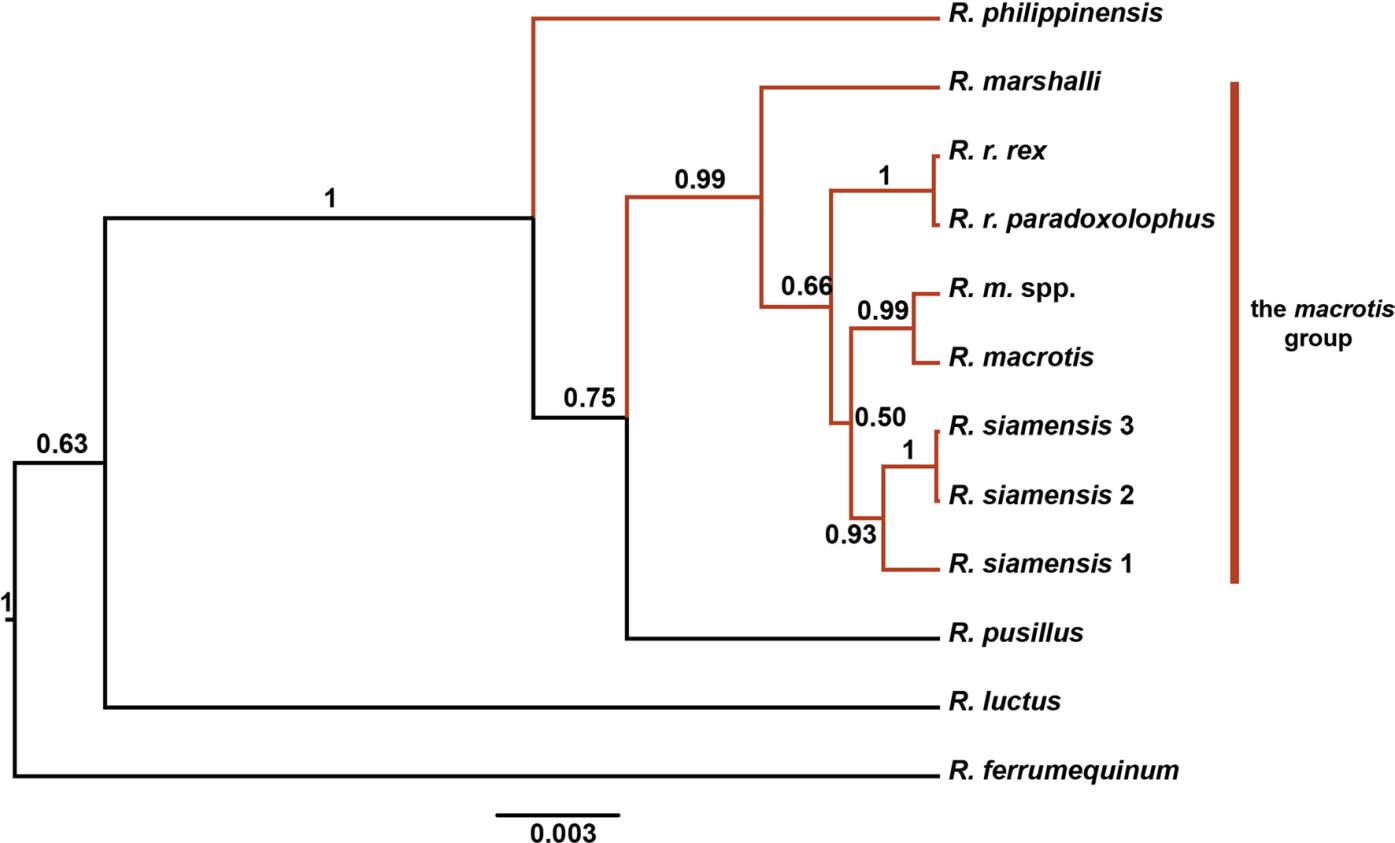
Phylogenetic tree of *Rhinolophus* species obtained in the former *philippinensis* group. Colored branches indicate bat species with low echolocation frequency relative to body size. This tree is reproduced from Zhang et al. [33]

Among the horseshoe bats (genus *Rhinolophus*), those in the *Rhinolophus macrotis* and *R. philippinensis* groups have lower echolocation frequencies than other rhinolophids with similar body sizes, except for *R. osgoodi* [32]. Our field observations have shown that species in these two groups have relatively low flight speeds compared with the flight speeds of other species. Evolutionary constraints on the mtDNA of birds were found to lead to the accumulation of more non-synonymous than synonymous substitutions in weakly flying and flightless birds compared with those accumulated in rapidly flying birds [22]. These unique phenomena may have a genetic and possibly adaptive basis, making the *philippinensis* and *macrotis* groups suitable for studies into the role of natural selection in determining specific traits. The *macrotis* group was separated from the former *philippinensis* group in a phylogenetic tree constructed by Zhang et al. [33] that included six closely related species; *R. macrotis* Blyth, 1844; *R. siamensis* Gyldenstolpe, 1917; *R. rex* Allen, 1923; *R. osgoodi* Sanborn, 1939; *R. marshalli* Thonglongya, 1973; and *R. schnitzleri* Wu & Thong, 2011 (Fig. 1). The *philippinensis* group now includes *R. philippinensis* Waterhouse, 1843; *R. montanus* Goodwin, 1979; and *R. achilles* Thomas, 1900. The *macrotis* and *philippinensis* groups are widely distributed across large parts of South and Southeast Asia, are morphology conserved, and have a young radiation history [33, 34]. The initial diversification of the *macrotis* group began during the Pleistocene and was potentially associated with the Tibetan Plateau uplift and climate changes during that time [35]. We speculate that both these groups may have undergone distinctive evolution, which suggests the possibility that natural selection acted at the mitogenome level. Although mitogenomes have been widely used in phylogenetic studies of many mammalian groups, most molecular phylogenetic studies of horseshoe bats have been based on limited numbers of mitochondrial genes [33, 36, 37]. No mitogenome-based phylogenic study of horseshoe bats has focused on the molecular evolution of energy metabolism-related genes, and thus their evolutionary history is poorly understood.

Only two mitogenomes of bat species with low echolocation frequency relative to body size (species_low_) are available in NCBI’s GenBank: *R. macrotis* (NC_026460) and *R. rex* (NC_028536). In this study, we sequenced eight mitogenomes including two additional species in the *macrotis* group as well as *R. philippinensis* and *R. pusillus* Temminck, 1834. We analyzed the newly sequenced complete mitogenomes and another four published *Rhinolophus* mitogenomes. We performed a comparative mitogenomes analysis for species_low_ to: (1) reveal characteristic and distinct nucleotide patterns; (2) reconstruct the mitochondrial phylogeny and assess the phylogenetic signals of single mitochondrial genes; and (3) detect signals of the adaptive evolution of the mitochondrial genes in genus *Rhinolophus*, especially those for species_low_ during their evolutionary processes.

## Material and methods

### Sampling and DNA extraction

Tissue samples were collected from eight individuals representing six species, then DNA was extracted and sequenced (detailed sampling information is provided in Additional file 1). Of the six species, three were *R. siamensis* considering that *R. siamensis* 2 and *R. siamensis* 3 were formerly recognized as *R. huananus* and *R.* cf. *siamensis*, respectively, and were considered as junior synonyms of *R. siamensis* [33] (Additional file 1). Three other species_low_ (*R. schnitzleri*, *R. achilles* and *R. montanus*) in the *macrotis* and *philippinensis* groups were not included in this study because of their limited distribution and sampling difficulty.

All samples were initially preserved in 100% ethanol in the field, then transferred and stored at − 80 °C until DNA extraction. Tissues were collected from muscle of voucher specimens or the wing membrane (biopsy punches: 3-mm diameter) of living bats. Total genomic DNA was extracted using a UNIQ-10 column animal genomic DNA isolation kit (Sangon Biotech, Shanghai, China), following the manufacturer’s instructions. To compare the patterns of mitogenome sequence evolution of the six species_low_, four rhinolophid mitogenome sequences from previously published studies were downloaded from NCBI (https://www.ncbi.nlm.nih.gov/), two of which are from the *macrotis* group (Additional file 2).

### PCR amplification and sequencing

We aligned the sequence of the 15 available complete rhinolophids mitogenomes from NCBI and the primers were designed in the conserved regions. The whole mitogenome of each sequenced individual was amplified in overlapping fragments with tens or hundreds of bases using 13 specific primers designed in Primer 5.0 [38] and Primer 6.0 [39]. The mitogenomes of *R. monoceros* Andersen, 1905 (NC_005433) and *R. macrotis* were used as reference sequences for primer design. We also designed specific primers for some regions that were not amplified using the 13 primers. The primer sequences used in this study are listed in Additional file 3. The PCRs and sequencing were conducted as described previously [33].

### Sequence annotation and bioinformatics analysis

Sequences were aligned and assembled using Geneious 9.0 [40]. The PCGs, rRNA and tRNA genes, and non-coding regions of each mitogenome were annotated using the complete mitogenome of *R. macrotis* as the reference sequence. The 13 PCGs were translated into amino acid sequences using the vertebrate mitochondrial genetic code and checked for unexpected frameshift errors or stop codons in MEGA v.7 [41]. Nucleotide composition was calculated, and total GC content and codon usage were analyzed. Strand asymmetry was calculated as follows: AT-skew = [A − T]/[A + T] and GC-skew = [G − C]/[G + C] [42]. DNA polymorphisms were calculated for each single gene using DnaSP v5.0 [43]. All the newly sequenced complete mitogenome sequences have been deposited in NCBI under Accession numbers MK987176–MK987183 as shown in Additional file 2.

### Phylogenetic analysis

Twelve mitogenome sequences, excluding intergenic and control regions, were used in the phylogenetic analyses (Additional file 2). The PCGs, rRNAs and tRNAs were aligned separately, and the 22 tRNAs were combined using SequenceMatrix [44] as one sequence because of the short sequence length for each tRNA. Then, all sites of 13 PCGs, 2 rRNAs and 22 tRNAs were combined as a concatenated dataset that contained 15,487 nucleotides. We used jModelTest 0.1 [45] to determine the best-fit nucleotide substitution models (Table 1) for the 13 PCGs, 12S rRNA, 16S rRNA, and the combined 22 tRNAs under the Akaike information criterion.

**Table 1:** Best substitution models for each of 13 protein-coding genes, and comparison of phylogenetic topologies recovered by different mitochondrial datasets

Gene	Model	The *macrotis* group	The *macrotis* complex	*R. r. rex* + *R. r. paradoxolophus*
*ATP6*	TrN + G	N	Y	Y
*ATP8*	TIM3 + I + G	N	N	Y
*Cytb*	TPM2uf + G	Y	Y	Y
*COX1*	TPM2uf + I + G	N	N	Y
*COX2*	HKY + G	N	N	Y
*COX3*	TIM2 + I	Y	N	Y
*ND1*	TIM2 + G	N	N	Y
*ND2*	TrN + I + G	N	N	Y
*ND3*	TrN + I	N	N	Y
*ND4*	TrN + I	Y	Y	Y
*ND4L*	TPM2uf + I	N	N	Y
*ND5*	TIM1 + G	Y	Y	Y
*ND6*	TIM3 + G	Y	Y	Y
*12S rRNA*	TPM2RF + I + G	N	N	Y
*16S rRNA*	TIM2 + G	N	N	Y
22 tRNAs	HKY + G	N	N	Y
Concatenated dataset		Y	Y	Y

Concatenated dataset: 13 protein-coding genes, 2 ribosomal RNA genes, and 22 transfer RNA genes. Three mitochondrial clades inferred from the mitochondrial concatenated dataset were defined

Y, phylogeny is supported; N, phylogeny is not supported

The phylogeny analysis was conducted using Bayesian inference with MrBayes 3.2.2 [46] and the concatenated dataset. To evaluate the phylogenetic signal for each single mitochondrial gene, single mitochondrial gene datasets with each of the 13 PCGs, 12S and 16S rRNAs, and the combined 22 tRNAs were used to reconstruct phylogenies. The Markov chain Monte Carlo technique was used to conduct two independent runs with four chains (two heated and two cold) simultaneously for 10 million generations, sampling every 1000 generations. After discarding the first 25% of the samples as burn-in, posterior probabilities were calculated in a consensus tree. Stationarity was assumed when the average standard deviation of split frequencies fell below 0.01. The resulting consensus tree was visualized in Figtree. We compared tree topologies obtained from each of the 16 single-gene datasets with the topology from the concatenated dataset.

### Analysis of selection pressure

#### Neutral evolution

We calculated the total number of amino acid replacements, the number of fixed non-synonymous substitutions and the number of synonymous changes for each gene using DnaSP.

According to methods used in Jacobsen et al. [65], all 13 PCGs were used to assess neutral evolution in species_low_ by calculating the linear regression between: (1) number of mutations and length in bases, (2) number of synonymous mutations and length in bases, and (3) number of non-synonymous changes and synonymous changes. The total number of amino acid replacements for each gene was corrected for gene length by calculating the number of amino acid replacements divided by the total number of amino acids in the gene.

#### Selection analysis

The selection level against non-synonymous substitutions relative to synonymous substitutions was measured as the ratio of the number of non-synonymous substitutions per non-synonymous sites (Ka) to the number of synonymous substitutions per synonymous sites (Ks). To compare the differences in sequence evolution pattern between species_low_ and other rhinolophids, the Ka/Ks distributions in pairwise comparisons between DNA sequences were calculated [47] for each of the 13 PCGs using Ka/Ks calculator [48]. We used SPSS 19 [49] to assess the significance of the Ka/Ks values.

Codon substitution has been widely used to detect adaptive signatures affecting protein evolution [50]. To identify signals of positive and purifying selection in the 13 PCGs for species_low_, three codon-based selection methods were applied: HYPHY, implemented in the Datamonkey server (http://www.datamonkey.org/) [51], CodeML in PAML v4.8 [52], and TreeSAAP version 3.2 [53]. Because each method is prone to false positives, we used multiple methods with different underlying assumptions and checked for congruence [10]. A species tree (Fig. 1) from Zhang et al. [33] was used in the selection analyses.

For the selection analyses of the 13 PCGs, stop codons were removed and the *ND6* sequence was reverse-complemented because *ND6* is on the light strand. Overlapping regions (7 bp of *ATP8*/*ATP6* and 4 bp of *ND4L*/*ND4*) were included in the alignment twice to enable analyses of all the codons.

##### Codon-based selection pressure (ω) ratio

Codons putatively under selection were detected using HYPHY. The FUBAR (Fast Unbiased Bayesian AppRoximation) [54] was used to detect codons under pervasive purifying or diversifying selection and MEME (Mixed Effects Model of Evolution) [55] was used to identify pervasive and episodic positive selection. The best-fit substitution model for each dataset was selected in HYPHY [56]. Significance was assessed by posterior probability (PP) > 0.9 (FUBAR) or *P* value < 0.05 (MEME).

CodeML, implemented in PAML, was used to run site and branch-site models by comparing a series of maximum-likelihood models to test for signatures of positive selection associated with particular codons or lineages. First, we used the free-ratio model, which allows ω to vary in different lineages. Then, we used the two-ratio model, which allows two sets of lineages to have different ω values, and used M0 (one ω ratio) to compare the lineages for foreground branches (branches for species_low_) with background branches (all remaining branches).

The site model allowed ω to vary among codons and assume one ω ratio for all branches. Six different site models were run using equal codon frequencies as proposed by Yang et al. [57]: M0 (one ω ratio), M1a (nearly neutral), M2a (positive selection), M3 (discrete), M7 (beta), and M8 (beta and ω). A likelihood ratio test (LRT) was used to compare a null model against a model that allowed codons to be ω > 1 as follows: M0 vs. M1a tested for a single ω ratio across codons, M0 vs. M3 assessed whether variable selection pressure was present among sites, and M1a vs. M2a and M7 vs. M8 tested presence of positive selection. When the LRT indicated that models predicted positive selection, a Bayes empirical Bayes (BEB) method implemented in the CodeML program of PAML was used to calculate the PPs of the codons that tested a ω > 1 in M2a and M8. PPs > 0.85 were considered as supported under the BEB method [57].

The CodeML branch-site model was also run to test whether some sites had undergone positive selection in a foreground branch of the phylogenetic tree. Signals of positive selection for codons on given branches were detected by considering the lineages of each species_low_ and the *macrotis* group as foreground branches. The alternative (MA, positive selection: 0 < ω_0_ < 1, ω_1_ = 1, ω_2_ ≥ 1) and null model (MA0, neutral evolution with ω_2_ = 1 fixed) were used to detect selective pressure on each branch. In all cases, an LRT was used for the pairwise model comparison and to determine which model better fits the data (*P* < 0.05). Positive selection was inferred when ω > 1 and the LRT was significant (*P* < 0.05). The BEB method was used to calculate PPs for site classes to determine which codon positions had experienced positive selections (ω > 1) on the foreground lineages [58].

##### Radical changes in physicochemical properties of amino acids

TreeSAAP was used to infer the direction of amino acid replacements by comparing mitogenomes codon-by-codon under the predefined phylogeny. TreeSAAP assigns weight values to non-synonymous codon changes based on the effects of amino-acid replacements on 20 physicochemical properties using the evolutionary tree (accuracy of detecting selection > 85%) [59]. We tested significant deviation from neutral evolution by calculating a z-score, and positive selection was assigned when the number of inferred amino acid replacements significantly exceeded the number expected by chance alone, yielding positive z-scores. The overall amino acid replacements are scored from 1 to 8, with eight as the most significant change. The analysis was run using a window size of 20 codons and sliding in 1-codon increment. To minimize false positives, only amino acid replacements with strong effects on protein biochemistry (z-score > 3.09 (corresponding to *P* < 0.001) and magnitude categories > 6) were considered to potentially be evolving under positive selection [60].

## Results

### Characteristics of the mitogenome

The complete mitogenomes sequenced in this study shared similar sequence characteristics. Each mitogenome was a closed-circular DNA molecule that contained a typical set of 37 genes: 13 PCGs, 22 tRNAs, 2 rRNAs, and a non-coding region. The gene arrangement, organization, and content of the mitogenomes were identical to those of other Chiroptera mitogenomes. The mitogenome ranged in size from 16,811 bp in *R. rex paradoxolophus* to 16,897 bp in *R. m.* spp. (sensu Tu et al. [61]) (Additional file 4). The length variation across different species was relatively low in the coding genes and the differences in length resulted largely from variations in the lengths of non-coding regions, especially in the control region. Except for *ND6* and eight of the tRNA genes, which were on the light strand, the other mitochondrial genes (PCGs, rRNAs, and other tRNAs) and the control region were on the heavy strand.

All these rhinolophid mitogenomes showed highly similar nucleotide composition biases (Additional file 2). All the mitogenomes were AT rich, with the AT content ranging from 56.2% (*R*. *siamensis* 3) to 57.1% (*R. philippinensis*). The AT content of the species_low_ mitogenomes was slightly lower than that of the other rhinolophids mitogenomes. All these mitogenomes were highly congruent in base skewness, with a positive AT-skew (0.099 to 0.111) and a markedly negative GC-skew (− 0.351 to − 0.321), suggesting strand heterogeneity in nucleotide composition.

### Mitochondrial phylogenetic analysis

#### Whole mitogenomic data

**Fig. 2:**
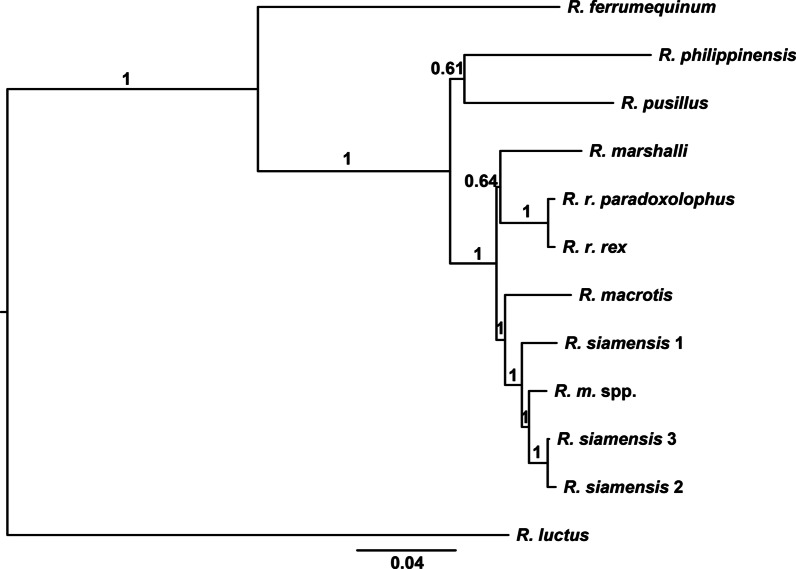
Phylogenetic tree of *Rhinolophus* species reconstructed using the concatenated dataset. The concatenated dataset contained the whole mitogenome sequences excluding the control region. The tree was constructed using Bayesian inference analyses in MrBayes. Posterior probabilities values are shown on the nodes

The molecular phylogeny reconstructed based on the concatenated dataset yielded topological relationships with high PPs (PP > 0.95 was considered as strong support in Bayesian inference) (Fig. 2). The topology supported the polyphyly for the former *philippinensis* group already noted by Zhang et al. [33]. Except for *R. philippinensis*, the other species in the former *philippinensis* group represented a monophyletic clade with high nodal support, named as the *macrotis* group in a previous study [33]. The *macrotis* group consisted of two sister clades: (1) the *macrotis* complex with robust statistical support and (2) *R. marshalli* + *R. rex* with a relatively low PP of 0.64.

#### Single mitochondrial genes

The alignment length and parsimony informative sites for each of the 13 mitochondrial PCGs are provided in Additional file 4. All phylogenies reconstructed based on 16 single-gene datasets showed different topologies (Additional file 5), and none of these topologies was identical to the phylogeny obtained with the concatenated dataset. Varied tree topologies among the different datasets indicated incongruent phylogenetic signals across the PCGs. The percentage of polymorphic sites varied among the 16 datasets, ranging from 10.51% in tRNAs to 31.65% in *ND4L* (Additional file 4). We compared the single-gene trees with the tree for the concatenated dataset in three clades (the *macrotis* group, the *macrotis* complex and *R. rex*, including the subspecies *R. r. paradoxolophus*) (Table 1). Six genes (*ATP6*, *Cytb*, *COX3*, *ND4L*, *ND5* and *ND6*) supported at least one of the three clades recovered by the concatenated dataset, whereas *Cytb*, *ND4L*, *ND5* and *ND6* supported all three clades.

### Analysis of selective pressure

#### Testing for neutral evolution

**Fig. 3:**
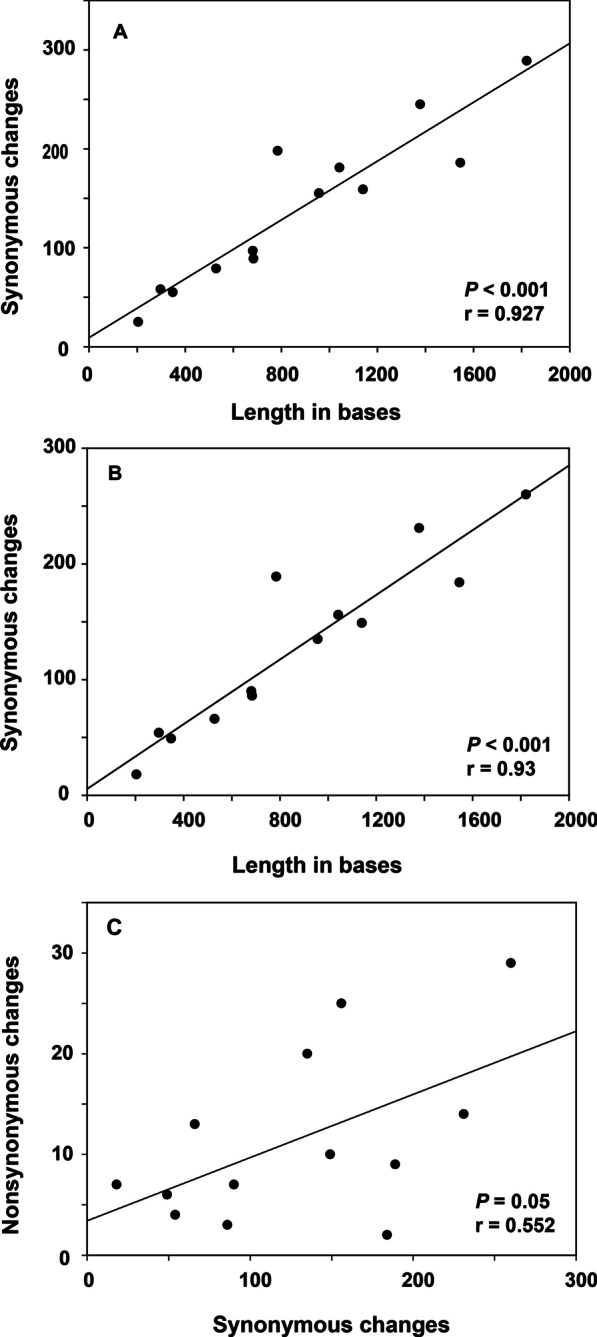
Correlation analyses. The 13 mitochondrial protein-coding genes of the bat species with low echolocation frequency relative to body size are indicated by dots. The lines are the best-fitted line. **A** Correlation between total number of mutations and gene length. **B** Correlation between synonymous mutations and gene length. **C** Correlation between non-synonymous and synonymous mutations. The correlation coefficient r and *P* value are shown in the lower right corner for linear regression.

The total number of mutations and the number of synonymous changes both showed significant linear relationships with gene length. The number of non-synonymous changes was correlated with the number of synonymous changes, although the correlation was not significant. Compared with the other genes, *ND2* and *ND5* showed relative high proportions of non-synonymous changes (Fig. 3).

#### Distribution of amino acid replacements across protein domains

**Fig. 4:**
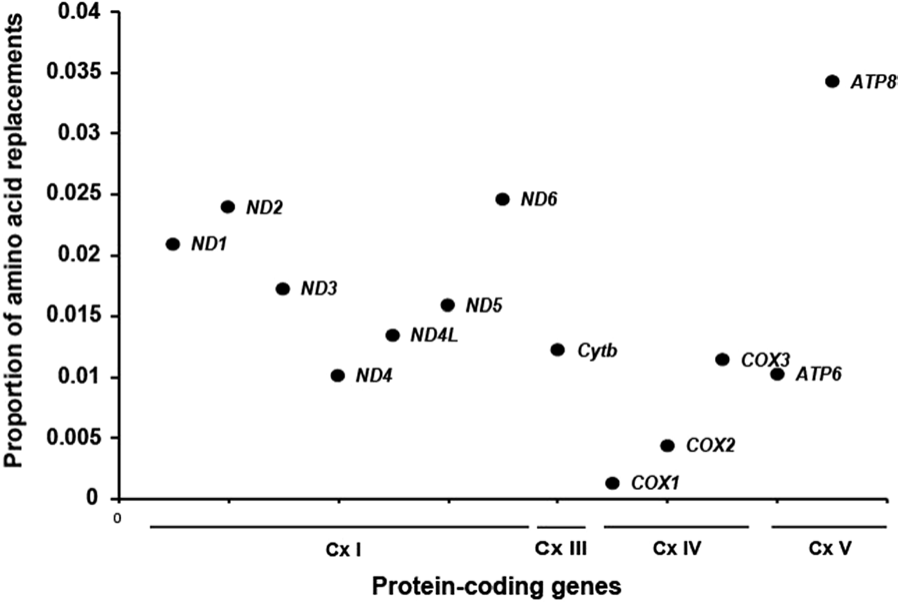
Fixed amino acid replacements in bat species with low echolocation frequency relative to body size. The proportion of amino acid replacements was calculated as the total number of replacements in each protein-coding gene divided by the gene length. The dots indicate the 13 different mitochondrial OXPHOS genes and the lines below the x-axis indicate the genes in the OXPHOS complexes (Cx)

The proportion of amino acid replacements, which was scaled by gene length, ranged from 0.001 (*COX1*) to 0.034 (*ATP8*) across 13 PCGs in species_low_ (Additional file 4). The proportions of amino acid replacements that were fixed for the NADH dehydrogenase genes ranged from 0.0101 (*ND4*) to 0.0246 (*ND6*), and that for *ATP8* (0.034) was higher than it was for the other genes (Fig. 4); these genes belong to OXPHOS complex I and complex V, respectively. Thus, complex I and complex V harbored a higher proportion of amino acid replacements than the other OXPHOS complexes.

#### Evidence of natural selection across all 13 mitochondrial protein-coding genes

##### Selective pressure on different species clusters

**Fig. 5:**
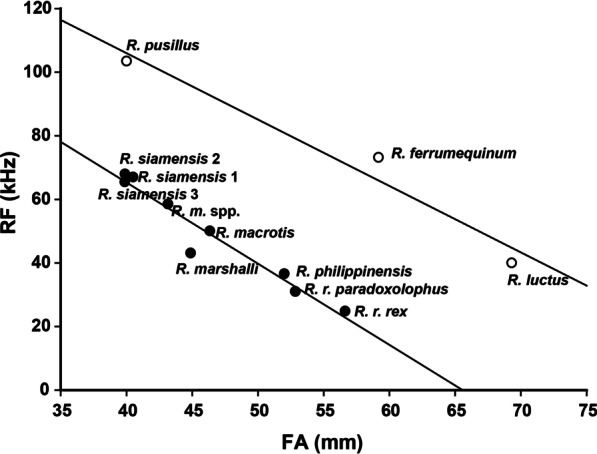
Relationship between average forearm length (FA) and resting frequency (RF) for the *Rhinolophus* species. Solid circles indicate species with low echolocation frequency relative to body size; empty circles indicate the other species

The species_low_ have lower echolocation frequencies than other species with similar body sizes (Fig. 5). Three genes (*ATP6*, *ND2*, and *ND5*) were found to have significantly different Ka/Ks values (*P* = 0.002, 0.018, 0.002, respectively) between the species_low_ and other species clusters (Table 2), indicating that the mitochondrial PCGs of the species in these two clusters experienced different evolutionary pressures.

**Table 2:** Non-synonymous/synonymous (Ka/Ks) values for 13 protein-coding genes of the species in two clusters

Gene	ka/ks_low_^a^	ka/ks_normal_^b^	*P* value^c^
*ATP6*	1.047779	0.012706	0.002**
*ATP8*	0.104115	0.080252	1
*COX1*	0.002006	0.00317	0.136
*COX2*	0.01567	0.013083	0.433
*COX3*	0.017378	0.017603	0.941
*Cytb*	0.024837	0.024236	0.501
*ND1*	0.038619	0.029385	0.823
*ND2*	0.050525	0.078366	0.018*
*ND3*	0.031403	0.032856	0.14
*ND4*	0.024325	0.016991	0.228
*ND4L*	0.031965	0.054756	0.092
*ND5*	0.036546	0.049335	0.002**
*ND6*	0.066561	0.08568	0.096

^a^Ka/Ks_low_, mean Ka/Ks values for the species that have lower echolocation frequencies than other species with similar body sizes

^b^Ka/Ks_normal_, mean Ka/Ks for the species in the other cluster

^c^Values in bold font indicate significant differences. Note: * 0.01 < *P *< 0.05, ** 0.001 < *P* < 0.01

The one-ratio model analyses showed that the ω values for the 13 PCGs were significantly < 1, suggesting that these *Rhinolophus* genes had experienced constrained selective pressure to maintain their functions.

The site model comparisons indicated that the mitogenome has evolved in a non-neutral pattern in *Rhinolophus* species. The LRT values for M0 vs. M3 were highly significant for eight genes (*ATP6*, *COX3*, *Cytb*, *ND1*, *ND2*, *ND3*, *ND4*, and *ND6*, *P* < 0.05) and M0 vs. M1a showed that eight genes had evolved non-neutrally (*COX3*, *Cytb*, *ND1*, *ND2*, *ND3*, *ND4*, *ND5*, and *ND6*, *P* < 0.05). These results indicate that the selection pressure (ω) for these genes varied among sites. No sites evolved under positive selection as indicated by M1a vs. M2a (LRT not significant). Two codons in *ND6* were under positive selection as identified by M7 vs. M8, and for one codon (*ND6*_101T_) the PP (PP = 0.99) was higher than the PP = 0.85 threshold according to the BEB analysis (Additional file 6, Table 3). The different codon numbers between the model comparisons are expected because M2a was a highly conservative test with lower power than M8 [23, 62, 63].

**Table 3:** Positive selected sites tested using different methods

Gene	Codon	Method	Parameter	Significant
*ND2*	331	Codeml (site model)	*ω*2 = 52.46859 (M2a) *ω* = 2.37570 (M8)	PP = 0.671 (M2a) PP = 0.809 (M8)
	331	FEL	*ω* > 1, LRT = 3.52	*P* = 0.06
	331	MEME	*ω* > 1, LRT = 4.22	*P* = 0.045*
*ND5*	543	Codeml (branch-site model)	*ω*2b = 40.98885	PP = 0.992**
	543	MEME	*ω* > 1, LRT = 14.45	*P* = 0.0003***
*ND6*	101	Codeml (site model)	*ω*2 = 6.32703 (M2a) *ω* = 6.63018 (M8)	PP = 0.925* (M2a) PP = 0.986* (M8)
	101	REL	Bayes factor = 981.166	PP = 0.98*

* indicate sites under positive selection. Note: * 0.01 < P < 0.05 or PP > 0.95, ** PP > 0.99, ***P < 0.001

The branch-site model was tested for each species_low_ and the *macrotis* group, and the results showed positive selection in four sites of *ND5* when *R. philippinensis* was set as the foreground branch, and the PP was high for only one site (*ND5*_543S_, PP = 0.992) (Additional file 6, Table 3). The MA vs. Ma0 and MA vs. M1a comparisons were significant for *ND5*_543S_. These results imply that this codon position in *R. philippinensis* experienced positive selection and accumulated a high number of non-synonymous mutations.

The results from HYPHY showed codons spread across all 13 PCGs with evidence of pervasive purifying selection within *Rhinolophus*. No codons involved in diversifying positive selection were detected by FUBAR, which suggested pervasive purifying selection occurred across all mitochondrial PCGs. MEME indicated two codons showed evidence of episodic positive selection in *ND2* and *ND5*, and these codons were also found in the site model (*ND2*_331I_, not significant) and branch-site model (*ND5*_543S_) implemented in PAML. The REL analysis identified one positively selected site in *ND6* (*ND6*_101T_, Bayes factor = 981.166, *P* = 0.98). Thus, PAML and HYPHY together suggested two amino acid replacements as candidates for positive selection (Table 3), and both sites supported by the two independent tests were in OXPHOS complex I. Another candidate site (*ND2*_331I_) which was significant in the MEME test, was also located in complex I.

##### Evolution of amino-acid physicochemical properties inferred by TreeSAAP

The results from TreeSAAP for the 13 PCGs of *Rhinolophus* suggested a prevalence of purifying selection and several significant physicochemical changes, which suggested positive selection. This result was similar to the result obtained with the codon models. We found that 19 of the 20 physicochemical properties were under purifying selection (global negative z-score < − 3.09, *P* < 0.001, Additional file 7). Evidence for positive selection was found for two physicochemical properties, with global z-scores above the significance threshold (z > 3.09, *P* < 0.001). The property equilibrium constant (ionization of COOH), which scored category level 8 (z = 27.4), and solvent accessible reduction ratio, which scored category level 7 (z = 5.3), indicated radical amino acid replacements had occurred by positive selection.

**Fig. 6:**
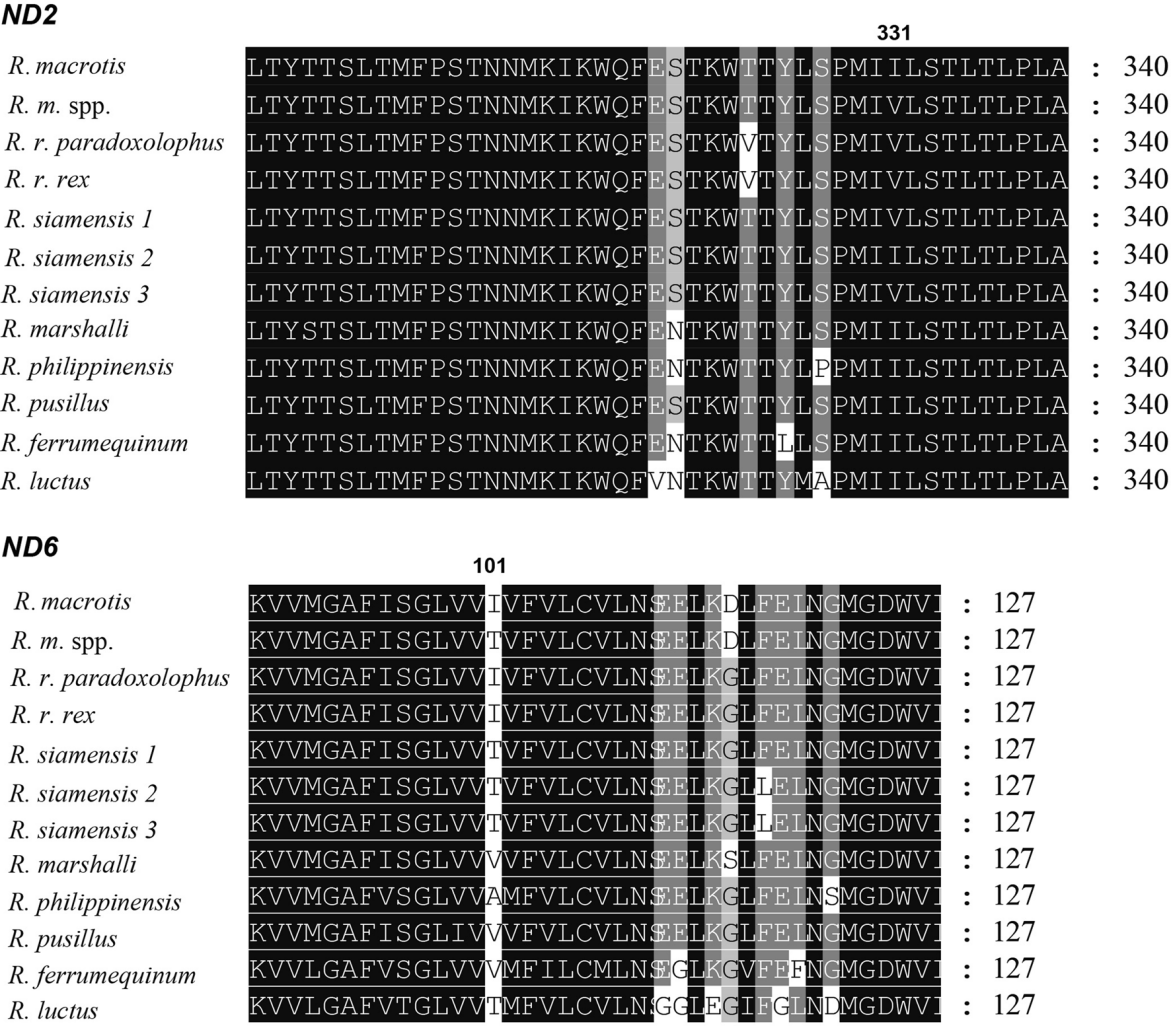
Multi-alignments of partial amino acid sequences encoded by *ND2* and *ND6* for the *Rhinolophus* species. Sites *ND2*_331I_ and *ND6*_101T_ are marked

The identified sites *ND2*_331I_ and *ND6*_101T_ were considered as possible loci of positive selection, which scored category level 8 and significant positive z-scores (z > 3.09, *P* < 0.001) only in radical changes suggested for the equilibrium constant (ionization of COOH). Notably, different methods found signatures of positive selection along with amino acid property changes in similar regions (*ND2*_331I_ and *ND6*_101T_) (Fig. 6). The replacement fixed within *ND5* (*ND5*_543S_) was supported as potentially under positive selection for buriedness (z > 3.09, *P* < 0.01) in TreeSAAP, but this was not significant.

## Discussion

In this comprehensive study, we present a comparative analysis of the mitogenomes of *Rhinolophus* species and examined natural selection at the molecular genetic level for species_low_. We sequenced eight individuals and included 12 mitogenomes in the analysis. The results showed that several codons and lineages were under positive selection.

### Characters of the mitogenomes of species_low_

Mammalian mitogenomes are typically 16 kb in size [30] and the mitogenome reported have similar sizes. The general features of the mitogenomes, including genome size, gene content, gene arrangement, codon usage, and secondary structures of the tRNAs, were highly conversed in species_low_, and similar to those of other *Rhinolophus* species. Analysis of variation in structure, nucleotide composition, codon bias, and other parameters among bats will improve our understanding of the evolutionary processes involved in their diversity. Therefore, we investigated the diversity in eight new bat mitogenomes to identify features associated with their evolution.

The mitogenome of *R*. *m. *spp. was found to be longer and the AT content of the mitogenomes of all the species in the *macrotis* group was lower than those of the other investigated *Rhinolophus* species, including *R. philippinensis*, a representative of a closely related species group. Because AT pairs have two hydrogen bonds and GC pairs have three hydrogen bonds, low AT content is considered more stable than high AT content, and therefore high AT content may lead to a less stable structure and possibly accelerated evolutionary adaptation. The lower AT content for the *macrotis* group may be an important adaptive characteristic, reflecting mitochondrial DNA variations and evolutionary adaptation.

### Phylogenetic analyses for species_low_

Mitochondrial genes have been widely used in studies of phylogeny, phylogeography, and evolution [1, 9, 10, 17, 23, 63]. Phylogenies obtained from complete mitogenomes represent the highest phylogenetic branch supports and therefore clearest taxonomic resolution [9]. Our phylogenetic analyses based on a concatenated dataset recovered a highly supported topology that was congruent with previous comprehensive phylogenetic analyses using a concatenated dataset of four mitochondrial genes for the former *philippinensis* group [33]. In this study, the polyphyly for the former *philippinensis* group and monophyly for the newly established *macrotis* group were well supported. The species in the *macrotis* group showed highly similar morphological characteristics, and clustered in a highly supported monophyletic clade, which was similar to the relationships obtained from the concatenated mitochondrial dataset in Zhang et al. [33]. *Rhinolophus m.* spp. clustered with *R. siamensis*, which was also found by Tu et al. [61] and Zhang et al. [33]. These findings combined with those of previous studies suggest that ancient introgression may exist because of climate fluctuations when bats diversified, and ancestral polymorphism was preserved because of the female philopatry and male-biased dispersal [33, 61]. *R. r. rex* and *R. r. paradoxolophus* clustered in one clade, which verified the species delimitation that *R. r. paradoxolophus* is a subspecies of *R. r. rex* as reported previously [32, 33]. *R. marshalli* clustered with *R. rex*, which is inconsistent with the results of the molecular study based on a concatenated multilocus dataset in Zhang et al. [33] but in accordance with the anatomical traits including the nasal foliation and cranial morphology [34].

Because of their linked nature, single mitochondrial genes are expected to carry the same phylogenetic signal as the whole mitogenome [9]. However, our results showed incongruent phylogenetic topologies among different genes and a different percentage of polymorphic sites in a single gene from different species, which implied the incongruent information was included in these genes. These findings may result from the varying evolutionary and selection pressure, as reported for other species [9, 13, 15].

Although recombination does not normally occur in mitogenomes [4], different regions have different evolutionary rates [9, 64–66], and we also observed this in the phylogenetic analyses. Several studies have indicated that phylogenies obtained from mitogenomes are better than those obtained from single genes, and the most informative mitochondrial genes that generated phylogenies similar to the whole mitogenome have been identified [9, 13, 67]. In this study, none of the single genes recovered the same topology as the concatenated dataset. However, we identified four genes (*Cytb*, *ND4L*, *ND5*, and *ND6*) that produced topologies similar to the concatenated mitogenome dataset in three defined clades.

Selection that causes differences in non-synonymous rates among mitochondrial genes is also important because it produces variable evolutionary rates that result from differences in the strength of purifying selection caused by functional constraints [66, 68]. Although the numbers of neutrally evolving synonymous mutations are highly correlated with the lengths of the corresponding genes (Fig. 3B), reflecting neutral evolution, the relative rates of non-synonymous mutations differ between genes (Fig. 3C). This is consistent with gene-specific differences in purifying selection.

### Analyses of selective pressures in the mitochondrial genes

We explored signatures of natural selection in the *Rhinolophus* mitogenomes. We detected a strong and widespread signature of ω < 1 across the 13 PCGs, which is evidence of purifying selection on these genes that have important functions in OXPHOS. Purifying selection appears to be the main evolutionary force that affects mitogenome evolution in bats. This result is consistent with the results of studies in other species such as fishes and mammals [3, 19]. Although the mitogenomes evolved under purifying selection to conserve the functionality of OXPHOS proteins [4, 68], we also found heterogeneous rates of ω across sites, and evidence of positive selection (ω > 1) in some codons. Positive selection in mitogenomes has been studied in a wide range of taxa, and has been associated with adaptation to extreme environments, size, and migration behaviour [9, 21, 22, 69]. Mitochondrial genetic variation was shown to affect ATP synthesis, mitochondrial content, and even the electrochemical gradient [70], suggesting the strongly potential role of mitogenomes in adaptation [18, 71].

The NADH dehydrogenase complex is the first and largest enzyme complex in the oxidative phosphorylation pathway in the mitochondrial electron transport chain [3, 4]. Previous studies on vertebrate species found positive selection signals in genes that encode proteins of OXPHOS complex I, including *ND2*, *ND5*, and *ND6* [3, 18, 23, 72, 73]. We found signatures of positive selection in *Rhinolophus* species according to the site model in CodeML and codon tests in HYPHY (Table 3). *ND6*_101T_ was found to have sites undergoing positive selection and changes in physicochemical properties occurred in the similar equilibrium constant (ionization of COOH) property for *ND2*_331I_ and *ND6*_101T_. The significant changes in the equilibrium constant (ionization of COOH) suggested adaptation to changing metabolic requirements has occurred [17]. Most physicochemical changes occur in regions genes that are likely under less conservative evolutionary constraints [65]. Genes that encode the NADH dehydrogenase subunits were less informative [9], and some of these genes were longer and/or had high percentages of polymorphic sites than other genes (Additional file 4), and therefore may be more likely to cause property changes. Our results are concordant with these findings.

*ND2* shows several patterns compatible with positive selection. *ND2* harbors a high percentage of the total amino acid changes and has relatively more non-synonymous substitutions than other genes in *Rhinolophus* mitogenomes (Fig. 3B). Moreover, the codon-based tests for positive selection in PAML (site model) and HYPHY (FEL) found little evidence of positive selection for *ND2*_331I_. Although ω > 1 was tested in the positive selection models, it was not significant in the model comparisons. However, MEME in HYPHY showed significance for codons *ND2*_323_ (*P* = 0.01) and *ND2*_331_ (*P* = 0.045), implying episodic diversifying selection occurred. The TreeSAAP results showed significant changes of in the equilibrium constant (ionization of COOH) property for *ND2*_331I_. Thus, we conclude that *ND2* was most likely under relaxed purifying selection. Jacobsen et al. [65] also proposed that relaxed purifying selection was driving the evolution of *ND2* by affecting mostly regions, included position 331, that have lower functional relevance. They also concluded codon 331 in *ND2* was under possible diversifying positive selection [65].

We did not find shared non-synonymous changes in all species_low_ (Fig. 6), but the Ka/Ks values of three genes were significantly different between species_low_ and the other species. In particular, *ATP6* (Table 2) had significantly higher Ka/Ks values in species_low_ than it had in the other species (Table 2), suggesting possible connections between codon changes and phenotype differences. For other flying animals such as insects and birds, adaptive evolution and more non-synonymous nucleotide substitutions accumulated in the mitochondrial genes of weakly locomotive animals [20, 22]. For species_low_ with relatively low flight speeds, their lower dispersal ability may result in lower effective population sizes, leading to a higher probability of reaching fixation for most nonsynonymous mutations [22, 74]. However, more evidence is needed to validate this conjecture.

Among the species_low_, we detected a signal of positive selection in *R. philippinensis*. *ND5*_543S_ was identified as a significant amino acid variation when the clade of *R. philippinensis* was set as the foreground lineage under the branch-site model in PAML. *R. philippinensis* is a young species with a distribution range across the Southeast Asian tropics [33, 34], and it is distributed allopatrically with other species_low_. Variation in mitochondrial OXPHOS genes may convey a signal of adaptation to the environment, particularly climatic conditions [63, 75–77]. Thus, ecological environment adaptation may lead *R. philippinensis* to be energetically different and undergoing natural selection that is different from species of the *macrotis* group. Further analyses and additional insights into the molecular adaptation of mitochondrial DNA in response to the tropical habitats of *R. philippinensis* are needed.

## Conclusion

Mitogenome studies have increased over the years, but, analyses of complete bat mitogenomes are still very rare. We obtained eight new complete mitogenomes representing species of the *macrotis* group, *R. philippinensis*, and *R. pusillus* to evaluate the molecular phylogeny and adaptive evolution. The known mitogenomes of the *Rhinolophus* genus vary in length from 16,775 bp (*R. luctus*) to 16,897 bp (*R.* m. spp.) and encode 37 genes, which have conserved general features. The phylogenetic topology reconstructed from the concatenated dataset corroborated the recently recognized *macrotis* group. The phylogenetic analyses results suggested the concatenated dataset performed better than single genes. Among the single genes, *Cytb*, *ND4*, *ND5*, and *ND6* were the most informative in the phylogenetic analyses. Selective pressure analysis demonstrated that complex I and complex V were under relatively strong purifying selection, whereas, for complex IV, purifying selection was relatively weak. Purifying selection is widespread in mitogenomes and it plays a major role in maintaining OXPHOS function during mitogenome evolution. We found evidence of positive selection in genes that encode proteins in complex I. *ND6* was shown to be under positive selection and *ND2* was inferred from codon tests to experience relaxed purifying selection in *Rhinolophus* species. *ND5* was shown to be under positive selection in *R. philippinensis*, suggesting ecological environment adaptation had occurred. Further work is needed to discover the molecular adaptation mechanism of mitochondrial DNA in the *Rhinolophus* genus.

## Supplementary Information

**Additional file 1: Table S1.** Voucher numbers of samples used in this study.

**Additional file 2: Table S2.** Length and base composition of each genomic component for species used in this study.

**Additional file 3: Table S3.** PCR primers used in this study.

**Additional file 4: Table S4.** Polymorphic sites of 13 PCGs, 2 rRNA genes and the combined 22 tRNA genes for species in this study, and the mutation number of each single PCGs in species_low_ is shown.

**Additional file 5: Figure S1.** Phylogenetic trees reconstructed from each single gene in MrBayes and posterior probability values are shown on the nodes.

**Additional file 6: Table S5. **Results of positive selection based on site model and branch-site model testing in protein-coding mitochondrial genes.

**Additional file 7: Table S6.** Significant values (*P* < 0.001) of TreeSAAP positive and negative z-score for amino acid physiochemical properties among radical categories 6, 7 and 8.

## Data Availability

The final complete mitogenome sequences sequenced in this manuscript are deposited in NCBI with Accession Number MK987176–MK987183.

## Abbreviations

Mitogenome: Mitochondrial genome
OXPHOS: Oxidative phosphorylation system
PCG: Protein-coding gene
ATP: Adenosine triphosphate
rRNA: Ribosomal RNA
PP: Posterior probability
FUBAR: Fast Unbiased Bayesian AppRoximation
MEME: Mixed Effects Model of Evolution
LRT: Likelihood ratio test
BEB: Bayes empirical bayes
