# Supplementary material for: Complete mitochondrial genomes reveal robust phylogenetic signals and evidence of positive selection in horseshoe bats
Source: BMC Ecol Evol. 2021 Nov 3;21:199. doi: 10.1186/s12862-021-01926-2 (PMC8565063; doi:10.1186/s12862-021-01926-2)
Supplement: Supplementary file 1 — Additional file 1: Table S1. Voucher numbers of samples used in this study. [file 12862_2021_1926_MOESM1_ESM.docx]

**Table S1.** Voucher numbers of samples used in this study.

| Species | Previous classification | Voucher | Tissue^a^ | Sex^b^ | Locality | Country |
| --- | --- | --- | --- | --- | --- | --- |
| *Rhinolophus siamensis* 1 |  | 48 | W | M | rain forest | China |
| *Rhinolophus siamensis* 2 | *Rhinolophus huananus* (Wu et al., 2008) | GD16002 | W | M | Zhaoqing | China |
| *Rhinolophus siamensis* 3 | *Rhinolophus* cf*. siamensis* (Tu et al., 2017) | HuN10111 | M | F | Lengshuijiang | China |
| *Rhinolophus macrotis* spp. | *Rhinolophus* cf*. macrotis* (Tu et al., 2017) | 121 | W | F | Jinning | China |
| *Rhinolophus marshalli* |  | GX07039 | W | M | Nanning | China |
| *Rhinolophus philippinensis* |  | CSOMA151 | W | F | Sarawak | Malaysia |
| *Rhinolophus rex paradoxolophus* | *Rhinolophus paradoxolophus* (Csorba et al., 2003) | 25003 | W | M | Xuan Lien NR | Vietnam |
| *Rhinolophus pusillus* |  | GZ17005 | M | F | Bijie | China |

^a^ M indicate tissues taken from muscles and W indicate tissues taken from wing membranes.

^b^ F indicate females for the corresponding samples and M indicate males.

Reference

1. Csorba G, Ujhelyi P, Thomas N. Horseshoe bats of the world (Chiroptera: Rhinolophidae). Bishop's Castle, UK: Alana Ecology Ltd. 2003.

2. Tu VT, Alexandre H, Tamás G, Satoru A, Dai F, Thanh HT, Son NT, Furey NM, Csorba G. Integrative taxonomy of the *Rhinolophus macrotis* complex (Chiroptera, Rhinolophidae) in Vietnam and nearby regions. Journal of Zoological Systematics and Evolutionary Research*.* 2017; 55, 177-198.

3. Wu Y, Motokawa M, Harada M. A new species of horseshoe bat of the genus *Rhinolophus* from China (Chiroptera: Rhinolophidae). Zoological Science. 2008; 25, 438– 443.
