# Supplementary material for: Complete mitochondrial genomes reveal robust phylogenetic signals and evidence of positive selection in horseshoe bats
Source: BMC Ecol Evol. 2021 Nov 3;21:199. doi: 10.1186/s12862-021-01926-2 (PMC8565063; doi:10.1186/s12862-021-01926-2)
Supplement: Supplementary file 2 — Additional file 2: Table S2. Length and base composition of each genomic component for species used in this study. [file 12862_2021_1926_MOESM2_ESM.docx]

**Table S2.** Length and base composition of each genomic component for species used in this study.

| Species | Size | A + T% | AT-skew | GC-skew | GenBank accession number | Reference |
| --- | --- | --- | --- | --- | --- | --- |
| *Rhinolophus macrotis* (Blyth, 1844) | 16,848 | 56.5 | 0.10376 | -0.32542 | NC_026460 | [Zhang *et al.* 2015](#_ENREF_4) |
| *Rhinolophus siamensis* 1 (Gyldenstolpe, 1917) | 16,846 | 56.5 | 0.106159 | -0.32679 | MK987183 | This study |
| *Rhinolophus siamensis* 2 (Gyldenstolpe, 1917) | 16,865 | 56.3 | 0.100863 | -0.3194 | MK987178 | This study |
| *Rhinolophus* *siamensis* 3 (Gyldenstolpe, 1917) | 16,822 | 56.2 | 0.101903 | -0.32165 | MK987177 | This study |
| *Rhinolophus* *macrotis* spp. (sensu: Tu et al., 2017) | 16,897 | 56.4 | 0.104606 | -0.32419 | MK987176 | This study |
| *Rhinolophus rex rex* (Allen, 1923) | 16,845 | 56.3 | 0.10195 | -0.31821 | NC_028536 | [Shi *et al.* 2015](#_ENREF_1) |
| *Rhinolophus rex paradoxolophus* (Bourret, 1951) | 16,811 | 56.4 | 0.103194 | -0.32114 | MK987180 | This study |
| *Rhinolophus marshalli* (Thonglongya, 1973) | 16,844 | 56.6 | 0.106191 | -0.32868 | MK987179 | This study |
| *Rhinolophus philippinensis* (Waterhouse, 1843) | 16,817 | 57.1 | 0.099354 | -0.32474 | MK987181 | This study |
| *Rhinolophus pusillus* (Temminck, 1834) | 16,858 | 57.3 | 0.10398 | -0.328 | MK987182 | This study |
| *Rhinolophus ferrumequinum* (Schreber, 1774) | 16,839 | 57.2 | 0.11113 | -0.3449 | NC_016191 | [Yoon *et al.*](#_ENREF_3) 2011 |
| *Rhinolophus luctus* (Temminck, 1834) | 16,775 | 58.6 | 0.10324 | -0.3508 | NC_018539 | [Xu *et al.* 2012](#_ENREF_2) |

**References**

1. Shi H, Zhang S, Mao X. The complete mitochondrial genome of the king horseshoe bat (*Rhinolophus rex*) using next-generation sequencing and Sanger sequencing. Mitochondrial DNA. 2015; 27, 2.

2. Xu H, Yuan Y, He Q*,* Wu Q, Yan Q, Wang Q*.* Complete mitochondrial genome sequences of two Chiroptera species (*Rhinolophus luctus* and *Hipposideros armiger*). Mitochondrial DNA. 2012; 23, 327.

3. Yoon KB, Kim JY, Cho JY, Park YC. The complete mitochondrial genome of the greater horseshoe bat subspecies, *Rhinolophus ferrumequinum* *korai* (Chiroptera: Rhinolophidae). Mitochondrial DNA. 2011; 22, 102-104.

4. Zhang L, Sun K, Feng J. Complete mitochondrial genome of the big-eared horseshoe bat *Rhinolophus macrotis* (Chiroptera, Rhinolophidae). Mitochondrial DNA. 2015; 27, 2.
