# Supplementary material for: Complete mitochondrial genomes reveal robust phylogenetic signals and evidence of positive selection in horseshoe bats
Source: BMC Ecol Evol. 2021 Nov 3;21:199. doi: 10.1186/s12862-021-01926-2 (PMC8565063; doi:10.1186/s12862-021-01926-2)
Supplement: Supplementary file 3 — Additional file 3: Table S3. PCR primers used in this study. [file 12862_2021_1926_MOESM3_ESM.docx]

**Table S3.** PCR primers used in this study.

| Primer name | Sequence (5'-3') |
| --- | --- |
| F12 | TTAACCAACCAAAGCAAGGCA |
| R1089 | GAAGTCTCCTGGGTGTAAG |
| F986 | TGAGAGGAGATAAGTCGTAAC |
| R5932 | GAAGAAGCACAGCCGTAA |
| F5782 | GCCATCTTCTCCCTTCATC |
| R7382 | GCTGAGGTCTTCGTAGTCTG |
| F7193 | CACAATAGACGCACAAGAAG |
| R9286 | GGAAGCATACGACAAGGAA |
| F9137 | GACTCTACTTCACTCTCCTAC |
| R12214 | ATAATGCCTACGCCTTCTC |
| F12038 | CGTAGCACTATTCGTCACA |
| R12480 | CCTCTATGGCTGATGGTA |
| F12190 | TCGGATGAGAAGGCGTAG |
| R12795 | CGGAGCATAGGAACAACAT |
| F12730 | CCCACCTAGCATTCCTTCA |
| R13470 | TTAGGGAGATTGATTTGGG |
| F12970 | CCTATACCAACGCCTGAGC |
| R14412 | TAGCGTAGAACTCAGCCGT |
| F14110 | GAATCTAACCACGACCAATG |
| R14675 | AGAATCCTCCTCAGACTCA |
| F14656 | ATGAGTCTGAGGAGGATTCT |
| R15412 | TGCTGATGGTGGAACTTG |
| F15124 | ACCTCTAAGCCAATGCCTA |
| R15525 | GAATAACATAGGGCGGGTGG |
| Pro | CAAGTTCCACCATCAGCACC |
| Phe | ACTCATCTAGGCATTTTCAGTG |
| F987 | TGAGAGGAGATAAGTCGTAAC |
| R3220 | GTAGTAGCATGGATAGTAGGAT |
| F980 | CGTAGTATGAGAGGAGATAAGT |
| R3183 | CTCGTAAGAGATGGTTTGTG |
| F3078 | ATCAAGCCTAGCCATATACTC |
| R3797 | GTTCGATTCCTGTGGTTCT |
| L5074 | CTGATAAAAGARTTACTTTGATAGAG |
| H6305 | GGCTTTGAAGGCYCTTGGTC |
| F4175 | GACCGTCACAAACATCCTA |
| R5269 | TATGCTACACGTTGAATTGC |
| F5004 | TCTAAGCAAATGAGCATAC |
| R5941 | ACAGGAGAAGAAGCACAG |
| F9110 | CCAAGCCCTGTTCATTAC |
| R10627 | TCCTGCATTCAGTCGTTC |
| F10512 | TAACATTCACCGCCACTG |
| R12216 | TATAATGCCTACGCCTTCTC |
