# Supplementary material for: Complete mitochondrial genomes reveal robust phylogenetic signals and evidence of positive selection in horseshoe bats
Source: BMC Ecol Evol. 2021 Nov 3;21:199. doi: 10.1186/s12862-021-01926-2 (PMC8565063; doi:10.1186/s12862-021-01926-2)
Supplement: Supplementary file 4 — Additional file 4: Table S4. Polymorphic sites of 13 PCGs, 2 rRNA genes and the combined 22 tRNA genes for species in this study, and the mutation number of each single PCGs in specieslow is shown. [file 12862_2021_1926_MOESM4_ESM.docx]

**Table S4.** Polymorphic sites of 13 PCGs, 2 rRNA genes and the combined 22 tRNA genes for species in this study, and the mutation number of each single PCGs in species_low_ is shown.

| Gene | Aligned length (bp) | Number of polymorphic sites | Percent of polymorphic sites (%) | Total number of mutation | Total number of replacement | Synonymous changes | Proportion |
| --- | --- | --- | --- | --- | --- | --- | --- |
| *ATP6* | 681 | 187 | 27.46 | 97 | 7 | 90 | 0.0102 |
| *ATP8* | 204 | 47 | 23.04 | 25 | 7 | 18 | 0.0343 |
| *COX1* | 1,545 | 388 | 25.11 | 186 | 2 | 184 | 0.0013 |
| *COX2* | 684 | 168 | 24.56 | 89 | 3 | 86 | 0.0044 |
| *COX3* | 784 | 190 | 24.23 | 198 | 9 | 189 | 0.0114 |
| *Cytb* | 1,140 | 289 | 25.35 | 159 | 14 | 145 | 0.0123 |
| *ND1* | 956 | 264 | 27.61 | 155 | 20 | 135 | 0.0209 |
| *ND2* | 1,042 | 294 | 28.21 | 181 | 25 | 156 | 0.0240 |
| *ND3* | 348 | 100 | 28.74 | 55 | 6 | 49 | 0.0172 |
| *ND4* | 1,378 | 393 | 28.52 | 245 | 14 | 231 | 0.0101 |
| *ND4L* | 297 | 94 | 31.65 | 58 | 4 | 54 | 0.0135 |
| *ND5* | 1,821 | 512 | 28.12 | 289 | 29 | 260 | 0.0159 |
| *ND6* | 528 | 142 | 26.89 | 79 | 13 | 66 | 0.0246 |
| 12S rRNA | 974 | 114 | 11.7 |  |  |  |  |
| 16S rRNA | 1,573 | 225 | 14.3 |  |  |  |  |
| 22 tRNAs | 1,532 | 161 | 10.51 |  |  |  |  |
