# Supplementary material for: Complete mitochondrial genomes reveal robust phylogenetic signals and evidence of positive selection in horseshoe bats
Source: BMC Ecol Evol. 2021 Nov 3;21:199. doi: 10.1186/s12862-021-01926-2 (PMC8565063; doi:10.1186/s12862-021-01926-2)

**Figure S1.** Phylogenetic trees reconstructed from each single gene in MrBayes and posterior probability values are shown on the nodes.


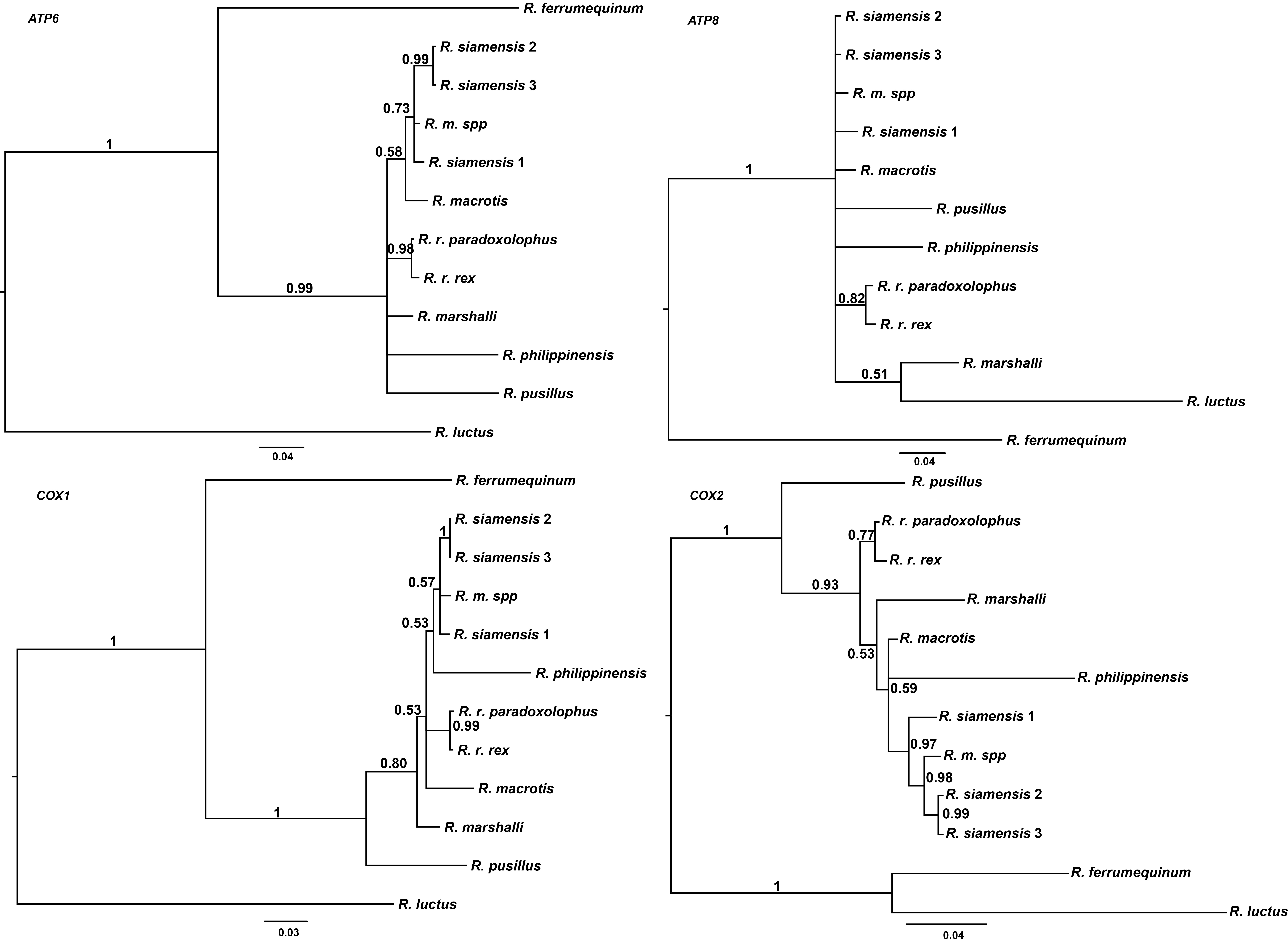


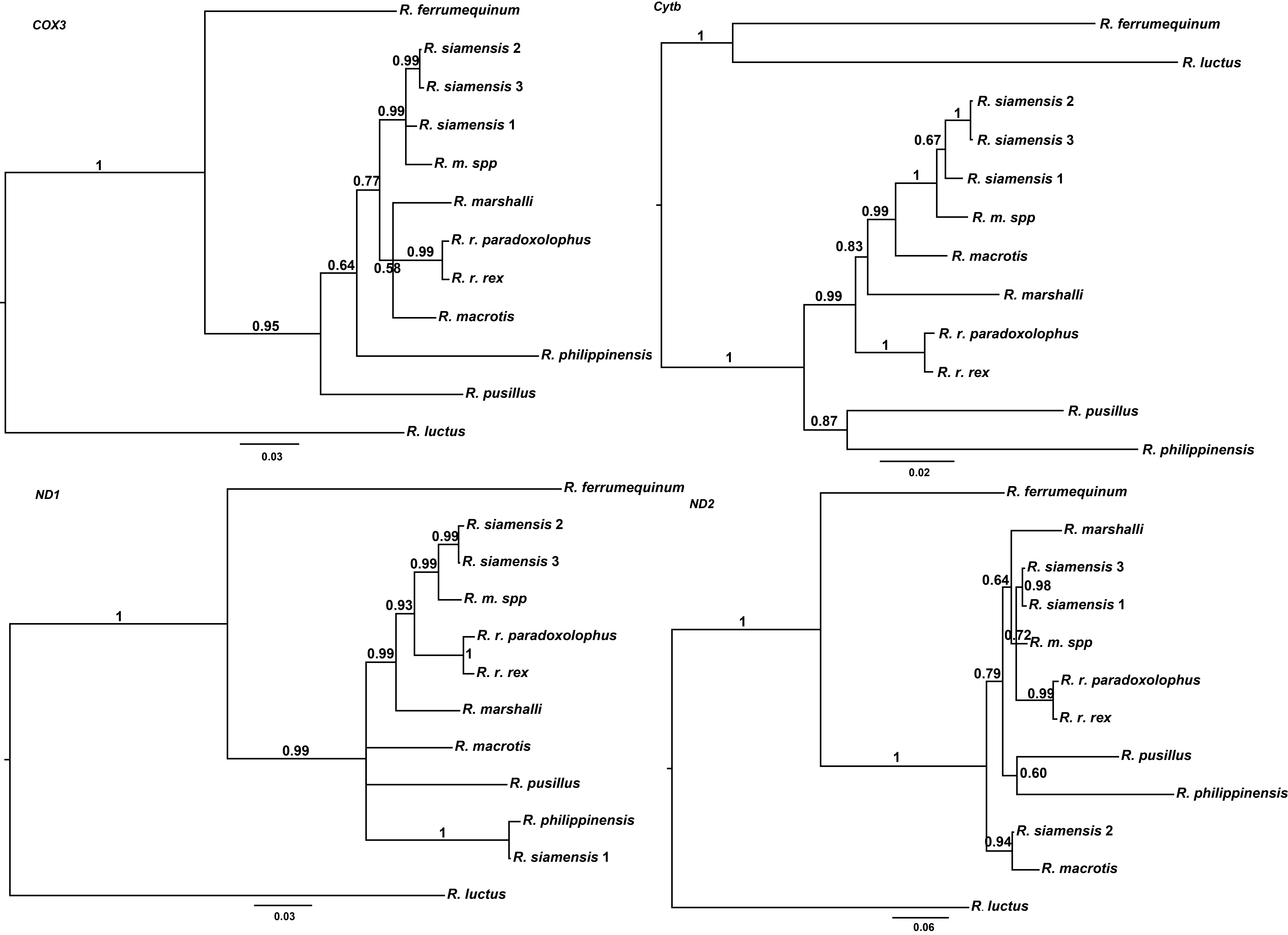


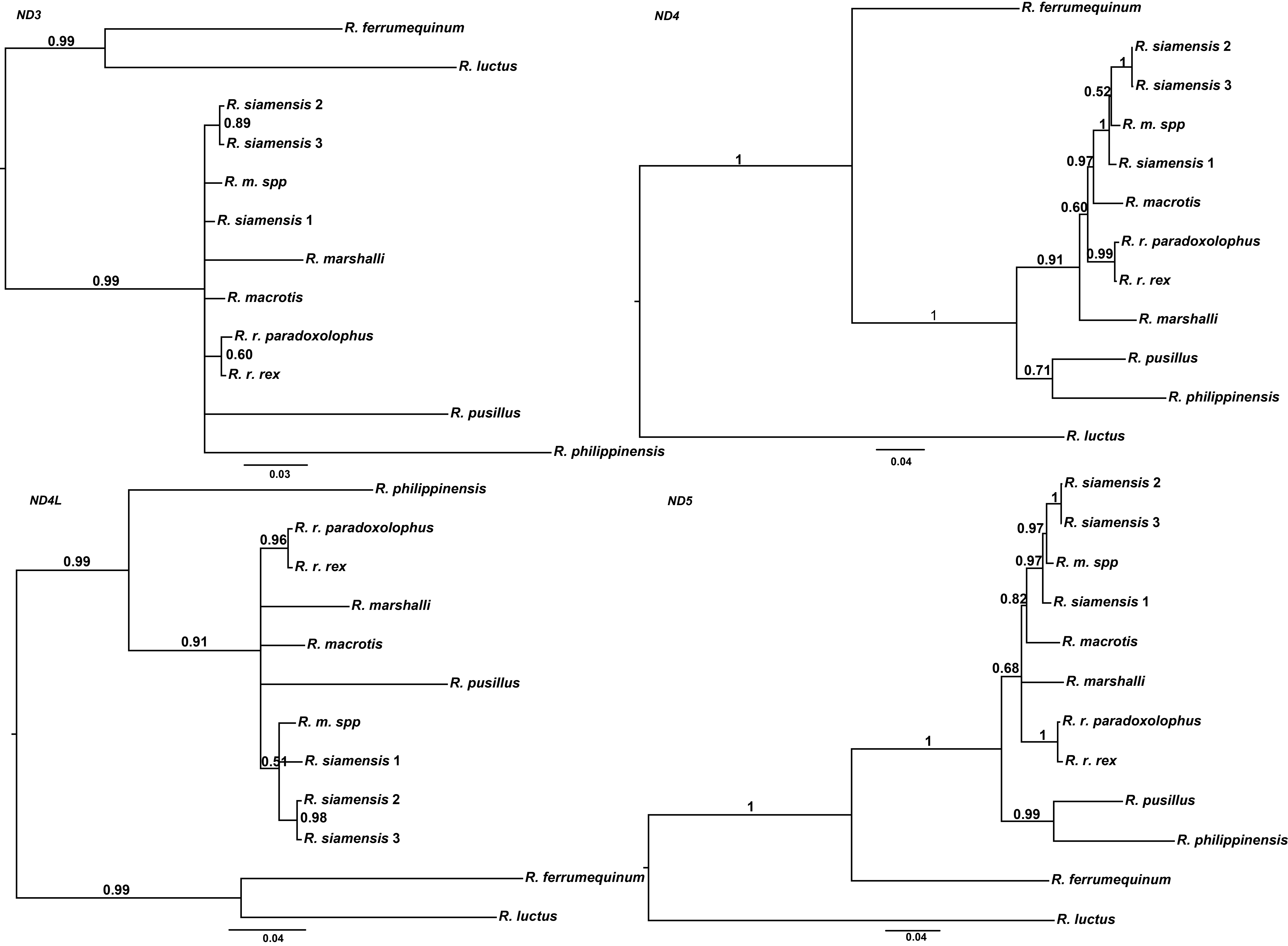


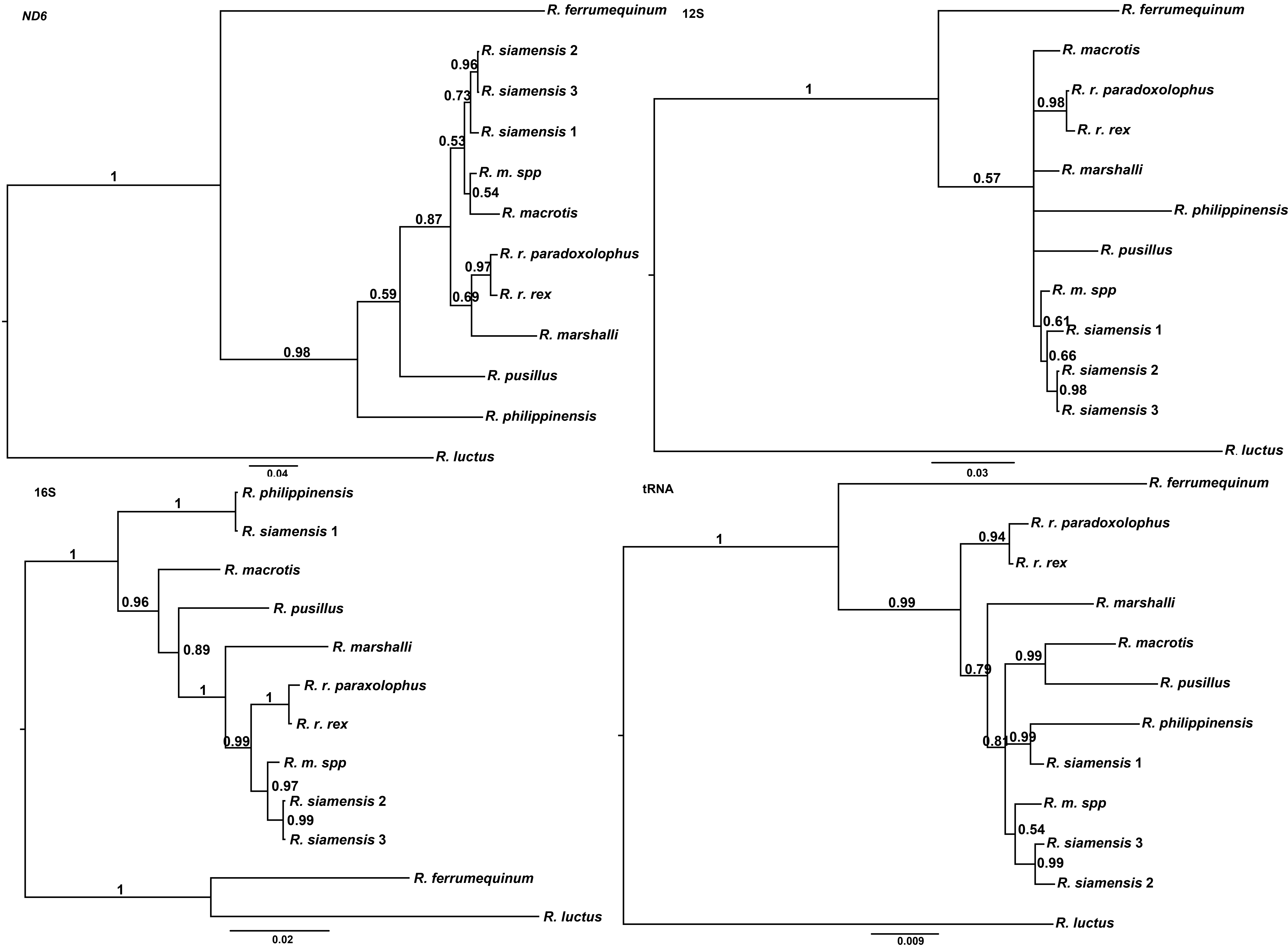

Supplement: Supplementary file 5 — Additional file 5: Figure S1. Phylogenetic trees reconstructed from each single gene in MrBayes and posterior probability values are shown on the nodes. [file 12862_2021_1926_MOESM5_ESM.docx]
