# Supplementary material for: Complete mitochondrial genomes reveal robust phylogenetic signals and evidence of positive selection in horseshoe bats
Source: BMC Ecol Evol. 2021 Nov 3;21:199. doi: 10.1186/s12862-021-01926-2 (PMC8565063; doi:10.1186/s12862-021-01926-2)
Supplement: Supplementary file 6 — Additional file 6: Table S5. Results of positive selection based on site model and branch-site model testing in protein-coding mitochondrial genes. [file 12862_2021_1926_MOESM6_ESM.docx]

**Table S5.** Results of positive selection based on site model and branch-site model testing in protein-coding mitochondrial genes.

| Gene | Model | Parameters | -lnL | Model test | LRT | *P* | Positively selected sites | |
| --- | --- | --- | --- | --- | --- | --- | --- | --- |
| *ND2* | M0 | *ω* = 0.03273 | 3294.62 |  |  |  |  | |
|  | M1a | p0 = 0.96234, (p1 = 0.03766), *ω*0 = 0.01856, (*ω*1 = 1) | 3271.90 | M1a vs M0 | 45.45 | <10^-3^ |  | |
|  | M2a | p0 = 0.96234, p1 = 0.03766, (p2 = 0.00000), *ω*0 = 0.01856, (*ω*1 = 1), *ω*2 = 52.46859 | 3271.90 | M2a vs M1a | 0 | 1.0 | 331I (0.671) | |
|  | M3 | *ω*0 = 0.00000, p0 = 0.62771, *ω*1 = 0.05485, p1 = 0.32028, *ω*2 = 0.40811, p2 = 0.05201 | 3264.53 | M3 vs M0 | 60.18 | <10^-3^ |  | |
|  | M7 | p = 0.13983, q = 3.09626 | 3265.28 |  |  |  |  | |
|  | M8 | p0 = 0.99946, (p1 = 0.00054), p = 0.14099, q = 3.15992, *ω* = 2.37570 | 3265.27 | M8 vs M7 | 0.01 | 0.99 | 331I (0.809) | |
| *ND6* | M0 | *ω* = 0.05410 | 1517.77 |  |  |  |  | |
|  | M1a | p_0_ = 0.96506, (p1 = 0.03494), *ω*0 = 0.03594, (*ω*1 = 1) | 1507.06 | M1a vs M0 | 21.42 | <10^-3^ |  | |
|  | M2a | p_0_ = 0.98161, p1 = 0.00924, (p2 = 0.00915), *ω*0 = 0.04138, (*ω*1 = 1), *ω*2 = 6.32703 | 1505.93 | M2a vs M1a | 2.26 | 0.32 | 101T (0.925), 116L (0.668) | |
|  | M3 | *ω*0 = 0.00000, p0 = 0.59324, *ω*1 = 0.00000, p1 = 0.13093, *ω*2 = 0.22753, p2 = 0.27583 | 1503.56 | M3 vs M0 | 28.42 | <10^-3^ |  | |
|  | M7 | p = 0.16506, q = 2.24106 | 1503.46 |  |  |  |  | |
|  | M8 | p_0_ = 0.99133, (p1 = 0.00867), p = 0.24055, q = 4.31972, *ω* = 6.63018 | 1499.73 | M8 vs M7 | 7.45 | 0.02 | 101T (0.986), 116L (0.744) | |
| *ND5* | M1a | p0=0.96085, p1=0.03915, *ω*0=0.01552 *ω*1=1 | 5347.77 | MA vs M1a | 15.45 | <10^-3^ |  |  |
|  | MA | p0=0.95681, p1=0.03837, p2a=0.00463, p2b=0.00019  Background *ω*: *ω*0=0.01446, *ω*1=1, *ω*2a=0.01446, *ω*2b=1  Foreground *ω*:*ω*0=0.01446, *ω*1=1, *ω*2a=40.98885, *ω*2b=40.98885 | 5340.04 | MA vs MA^’^ | 6.89 | 0.009 | 16T (0.774) 184M (0.815) 543S (**0.992**) 606E (0.575) |  |
|  | MA^’^ | p0=0.94478, p1=0.03620, p2a=0.01832, p2b=0.00070  Background *ω*: *ω*0=0.01444, *ω*1=1, *ω*2a=0.01444, *ω*2b=1  Foreground *ω*: *ω*0=0.01444, *ω*1=1, *ω*2a=1, *ω*2b=1 | 5343.49 |  |  |  |  |  |
