# Supplementary material for: Complete mitochondrial genomes reveal robust phylogenetic signals and evidence of positive selection in horseshoe bats
Source: BMC Ecol Evol. 2021 Nov 3;21:199. doi: 10.1186/s12862-021-01926-2 (PMC8565063; doi:10.1186/s12862-021-01926-2)
Supplement: Supplementary file 7 — Additional file 7: Table S6. Significant values (P < 0.001) of TreeSAAP positive and negative z-score for amino acid physiochemical properties among radical categories 6, 7 and 8. [file 12862_2021_1926_MOESM7_ESM.docx]

**Table S6.** Significant values (*P* < 0.001) of TreeSAAP positive and negative z-score for amino acid physiochemical properties among radical categories 6, 7 and 8.

| Physicochemical property | Positive | | | Negative | | |
| --- | --- | --- | --- | --- | --- | --- |
|  | 6 | 7 | 8 | 6 | 7 | 8 |
| Alpha-helical tendencies |  |  |  |  |  | -4.208 |
| Average number of surrounding residues |  |  |  |  |  | -5.001 |
| Buriedness |  |  |  |  | -6.373 |  |
| Chromatographic index |  |  |  | -5.717 | -5.756 |  |
| Coil tendencies |  |  |  |  | -4.257 | -6.378 |
| Compressibility |  |  |  | -4.072 | -3.463 |  |
| Equilibrium constant (ionization of COOH) |  |  | 27.408 |  |  |  |
| Helical contact area |  |  |  | -3.483 |  | -3.47 |
| Hydropathy |  |  |  |  | -5.066 | -5.964 |
| Isoelectric point |  |  |  | -7.742 | -3.311 |  |
| Mean r.m.s. fluctuation displacement |  |  |  | -6.324 | -6.033 |  |
| Normalized consensus hydrophobicity |  |  |  | -3.837 | -3.647 | -4.342 |
| Polarity |  |  |  | -3.282 | -6.652 | -5.074 |
| Power to be at the N-terminal |  |  |  |  | -3.453 | -3.119 |
| Short and medium range non-bonded energy |  |  |  |  | -3.553 | -4.981 |
| Solvent accessible reduction ratio |  | 5.331 |  | -4.758 |  | -4.425 |
| Surrounding hydrophobicity |  |  |  |  | -4.82 |  |
| Thermodynamic transfer hydrohphobicity |  |  |  | -5.112 |  |  |
| Total non-bonded energy |  |  |  | -6.247 | -5.922 |  |
| Turn tendencies |  |  |  |  | -5.109 | -5.546 |
